# Health-promoting behaviors in older adulthood and intrinsic capacity 10 years later: the HUNT study

**DOI:** 10.1186/s12889-024-17840-3

**Published:** 2024-01-24

**Authors:** Aslaug Angelsen, Sigrid Nakrem, Ekaterina Zotcheva, Bjørn Heine Strand, Linn Beate Strand

**Affiliations:** 1 Norwegian University of Science and Technology, Trondheim, Norway; 2 The Norwegian National Centre for Ageing and Health, Tønsberg , Norway; 3 Norwegian Institute of Public Health, Oslo, Norway

**Keywords:** Intrinsic capacity, Health-promoting behaviors, Public health, HUNT

## Abstract

**Background:**

With the global population growing older, there is a need for more knowledge of how to improve and/or maintain functional capacities to promote healthy ageing. In this study we aimed to assess the effect of several known health-promoting behaviors in old age with intrinsic capacity ten years later.

**Methods:**

This was a prospective cohort study looking at participants that were ≥ 65 years at the time of the third wave of the Trøndelag Health Study (HUNT3, 2006–2008) who also took part in the 70 + sub-study of the fourth wave (HUNT4 70+, 2017–2019). Self-reported behavior data from short questionnaires, including diet and physical activity, were collected in HUNT3, and data on the five domains of intrinsic capacity defined by the World Health Organization were collected in HUNT4 70+. A composite index was created for both healthy life and intrinsic capacity, awarding points for how well participants adhered to guidelines for healthy living and their level of functional impairment, respectively. Ordinal logistic regression was used to assess the relationship between health-promoting behaviors and intrinsic capacity.

**Results:**

Of 12,361 participants in HUNT3 ≥ 65 years, 4699 (56.5% women) also participated in HUNT4 70+. On the health-promoting behaviors, lowest adherence to healthy living guidelines were seen for fruit and vegetables intake (47.2%), milk intake (46.7%) and physical activity (31.1%). On intrinsic capacity domains, highest impairment was seen in the domains of locomotion (29.7%), hearing (11.1%) and vitality (8.3%). A higher adherence to guidelines for healthy living was associated with higher intrinsic capacity 10 years later. A one-point increase in the healthy life index was associated with a 1.15 (95% confidence interval 1.10–1.21) times increased odds of being in a higher intrinsic capacity category.

**Conclusion:**

Health-promoting behaviors in old age are associated with better intrinsic capacity ten years later. In clinical settings assessment of health-promoting behaviors could potentially be done using short questionnaires.

**Supplementary Information:**

The online version contains supplementary material available at 10.1186/s12889-024-17840-3.

## Introduction

Traditionally, health research and healthcare services have focused on the identification and treatment of disease, while the development and distribution of knowledge about how to improve and/or maintain functional capacities during ageing is limited [1, 2]. Except for the decrease in life expectancy due to the Covid-19 pandemic [3], life expectancy has increased in recent years while years of life lived in good health has not increased at the same rate [4].

The global population aged ≥ 65 years is projected to increase from 9.7% in 2022 to 16.4% in 2050 [5] (p8), therefore it is important to follow up on the World Health Organization (WHO) summons to invest in data to monitor healthy ageing across the life course [6]. According to the WHO, healthy ageing is the process of maintaining functional abilities to enable well-being in older age [6]. A helpful tool in this transition is the concept of “intrinsic capacity”, defined as an individual’s mental and physical capacities, further divided into the five domains locomotion, cognition, vitality, mental health and sensory. Intrinsic capacity was coined by WHO in 2015 [7], and in 2017 the first of WHO’s “Integrated Care for Older People” (ICOPE) guidelines were published. These guidelines provide a framework for screening, assessment and management of decline in intrinsic capacity.

Interventions suggested in ICOPE include nutrition and physical activity counselling, self-care and management skills and treatment of individual health conditions. Interventions should not be used in isolation, but rather “considered and applied together” [8]. Indeed, ICOPE behavior interventions mimic general country guidelines for healthy living that also include multiple modifiable behaviors to improve the healthiness of the population. Prior studies have shown that the number of health-promoting behaviors of an individual is associated with improved functioning in old age in the intrinsic capacity domains locomotion [9, 10], cognition [10, 11] and mental well-being [10, 12]. On the same note, health-promoting behaviors have been associated with lower mortality [13], less disability [13] and improved quality of life of older adults [14].

There is increasing awareness of the inadequacies of guidelines for treating single diseases, as individuals may present with multiple conditions that occur together [15]. Similarly, functional decline may occur in more than one domain of intrinsic capacity. Visser et al. [10] show the benefit of not only looking at multiple health-promoting behaviors together, but also including multiple functional domains when assessing the effect of health-promoting behavior on intrinsic capacity.

In this study we aimed to examine if multiple behaviors known to improve health in individuals ≥ 65, such as dietary habits and physical activity, were associated with improved intrinsic capacity at follow-up ten years later.

## Methods

### Study population

The Trøndelag Health Study (HUNT) is a longitudinal cohort study where self-report and clinical examinations have provided data on health, health related behavior, symptoms, illnesses, diseases and demographics from Mid-Norway [16]. Starting with the first data collection in 1984–1986, four waves have been performed with approximately 10 years between follow-ups. In HUNT3 (2006–2008) 50 807 participated (54.1% of the invited). The HUNT3 study cohort has been described in detail elsewhere [16]. With a larger focus on health in older age, in the fourth wave of HUNT all 19,403 participants that were 70 years or older were invited, whereof 9930 (51.2%) participated for additional clinical examinations (HUNT4 70+). The HUNT cohort is considered representative of the Norwegian population, except for a lack of representation of a larger city in the catchment area for the first three studies [16].

Our study cohort consists of individuals that were 65 years or older when participating in HUNT3 that also took part in the additional clinical examination in HUNT4 70 + 10 years later.

### Healthy life index

A composite healthy life index (HLI) was created using seven life factors known to influence health that was measured through participant self-report in HUNT3: Physical activity; social interaction; smoking status; sleep quality and dietary intake of fatty fish, fruit and vegetables, and intake of saturated fat from dairy and cooking fat. An equal contribution of each life factor to intrinsic capacity later in life was assumed. For physical activity and dietary intake, the Norwegian food based dietary guidelines recommendations were used to assess adherence to health-promoting behavior [17]. These guidelines contain multiple recommendations for food intake, and a single recommendation of beneficial levels of physical activity. Our index is dominated by dietary factors as intake of multiple food items included in the guidelines were measured in HUNT3, and we wanted to examine if the different recommendations had different effects on intrinsic capacity. Other recommended healthy habits such as a low intake of alcohol were also measured but not included in the summary score. Alcohol was excluded from the summary score due to the known association of people with chronic illness having a low intake, making it look like a higher intake of alcohol is beneficial when confounding is not taken into account [18]. While not included in the summary score, intake of alcohol was still analyzed as a stand-alone factor as it is known to influence health outcomes.

For each component included in the HLI participants could be given a maximum score of 1 for each factor considered no/mild risk to health, giving a maximum total score of 9 for the HLI. Similar to the work by Li et al. [19], each variable was categorized into “High”, “Moderate” or “No/low” risk of negative health outcomes. Participants could receive a score of 0, 0.5 or 1 based on the level of risk associated with the healthy life factor. The scoring system is detailed in Table 1, while each component of the HLI is explained in more detail in the supplementary files.

**Table 1:** Operationalisation of the healthy life index, based on variables from HUNT3

Behavior	Health-promoting behavior	Categorisation	Score
**Physical activity**	> 150 min moderate activity/week	< 75 min	0
		≥ 75 to < 150 min	0.5
		≥ 150 min	1
**Social interaction**	Not experience loneliness	Often lonely	0
		Sometimes lonely	0.5
		Not lonely	1
**Smoking**	Currently not smoking	Smoker	0
		Former smoker	0.5
		Non-smoker	1
Alcohol intake*	≤ Two units/week	≥ 7 units	0
		> 2 to ≤ 6 units	0.5
		≤ 2 units	1
**Sleep quality**	Not experience insomnia	Often experience insomnia Evening, night, morning	0
		Often experience insomnia Two timepoints	0.5
		Seldom/never experience insomnia	1
**Dietary factors**	Five portions of fruit and vegetables/day	< 2 servings	0
		≥ 2 to < 4 servings	0.5
		≥ 4 servings	1
	Two or more servings fatty fish/week	< 1 serving	0
		≥ 1 to < 2 servings	0.5
		≥ 2 servings	1
	Two glasses milk/day	< 1 glass	0
		≥ 1 to < 2 glasses	0.5
		≥ 2 glasses	1
	Choose low fat dairy products	Drink mostly whole milk	0
		Drink equal amounts whole and low fat milk	0.5
		Drink mostly low fat milk	1
	Choose oils or soft margarine as cooking fat	Use butter or hard margarine on bread and when cooking	0
		Use butter or hard margarine on bread or when cooking	0.5
		Use soft margarine or oils on bread and when cooking	1

*: Not part of the summary Healthy Life Index, but analyzed as a stand-alone factor

### Intrinsic capacity index

An intrinsic capacity index (ICI) was created using five variables measured in HUNT4 and HUNT4 70 + that fit the five domains of intrinsic capacity as defined by WHO [7]: Locomotion, cognition, vitality, psychology and sensory capacity. Sensory capacity was measured for both auditory and visual impairment. In each domain, participants could be classified as having a “High”, “Moderate” or “No/mild” impairment, corresponding to a score of 0, 0.5 and 1 respectively. The maximum score of the ICI was six, with a higher score corresponding to a higher capacity. Answers were either self-report or from performance tests. The scoring system is detailed in Table 2, while each component of the ICI is explained in more detail in the supplementary files.

All performance tests were done by trained personnel.

**Table 2:** Operationalisation of the five domains of intrinsic capacity based on variables from HUNT4 and HUNT4 70+

Domain	Measurement		Categorisation	Score
**Locomotion**	Short Physical Performance Battery		High impairment SPPB ≤ 6	0
			Moderate impairment SPPB ≤ 9	0.5
			No/mild impairment SPPB ≥ 10	1
**Cognition**	Montreal Cognitive Assessment		High impairment z-score ≤ -2	0
			Moderate impairment z-score ≤ -1	0.5
			No/mild impairment z-score ≥ -1	1
**Vitality**	Grip strength (kg)	Women	High impairment < 16 kg	0
			Moderate impairment < 19.9 kg	0.5
			No/mild impairment ≥ 20 kg	1
		Men	High impairment < 26 kg	0
			Moderate impairment < 31.9 kg	0.5
			No/mild impairment ≥ 32 kg	1
**Psychology**	Self reported life satisfaction		High impairment Very dissatisfied	0
			Moderate impairment Somewhat dissatisfied	0.5
			No/mild impairment Satisfied	1
**Sensory impairment**	Self reported sensory impairment		High impairment High impairment	0
			Moderate impairment Moderate impairment	0.5
			No/mild impairment No/mild impairment	1

### Potential confounders

#### Sociodemographic factors

Throughout HUNT, several sociodemographic factors are collected about participants that were controlled for in this study. We included categorical values for sex (Female and Male), level of education (Primary school/≤10 years, Vocational education/12 years, Upper secondary education/13 years, University or community college less than four years/<17 years, University or community college more than four years/≥17 years), marital status (“Married” and “Unmarried/Separated/Divorced/Widowed”, about 70% of those not married were widowed) and a continuous age variable.

#### Health factors

Participants were asked (“yes”/“no”) if they have had or were currently living with several long-term conditions: Diabetes, cancer, asthma, chronic obstructive pulmonary disease, renal failure, rheumatoid arthritis, fibromyalgia, stroke, cardiac arrest, heart failure, angina pectoris and “other heart conditions”. Living with long-term non communicable diseases is associated with a lower gain in healthy life expectancy [20], and is associated with fewer healthy behaviors [21]. To allow for as many levels of the variable as possible while not violating the proportional odds assumption of the ordinal logistic model, a four-level categorical variable was created based on the number of long-term conditions a participant had: 0, 1, 2 or 3+.

### Statistical analysis

All analyses were performed in R v. 4.2.2 [22]. and RStudio v. 2023.6.0.421 [23].

#### Descriptive statistics

Prevalence of level of risk from the health-promoting behaviors from HUNT3 and level of impairment from HUNT4 70 + was calculated and reported by sociodemographic characteristics and per quantile of the risk and impairment categories for the HLI and ICI respectively.

#### Missing data

Missing data were imputed using the multiple imputation framework from the R library mixgb v.1.0.1 with default settings [24]. Mixgb utilise XGBoost gradient trees, subsampling and predictive mean matching to impute variables. XGBoost is well known for its ability to accurately and efficiently capture complex data structures, while subsampling and predictive mean matching allow for better incorporation of the variability of missing data [25]. Before imputation, variables with > 20% missing data were removed. The percentage of missing data is reported with the risk and impairment categories.

#### Ordinal logistic regression

Ordinal logistic regression models were built to assess the relationship between health-promoting behaviors as an older adult and intrinsic capacity ~ 10 years later, using the polr function from the R library MASS v.7.3–57 [26]. An ordinal logistic regression model is well suited to our data, as level of impairment is ordered from lowest to highest impairment but the interval between any two points may not be equivalent [27].

Intrinsic capacity was categorised into five groups based on the summary score; ≤2, 3, 4, 5 and 6, with the score being rounded up to the largest number before categorisation, i.e. 2.5 would be “3”, and 5.5 would be “6”. Scores 1–2 were combined as there were only 18 participants that scored between 0 and 1.5. For the model using the HLI as an exposure, intrinsic capacity was categorised into four groups; ≤2, 3, 4 and ≥5, to fulfill the proportional odds assumption. Scores 5–6 were combined as it was assumed that combining the least impaired participants would preserve the most information from the different levels in the scale. Potential confounders were chosen based on previous literature and available variables in HUNT, and all models were adjusted for potential sociodemographic and health status confounders visualised in the directed acyclic graphs found in the supplementary material figures. Model 1 was adjusted for sociodemographic factors and health status confounders only, see Supplementary Fig. 1. Model 2 was additionally adjusted for other healthy behaviors as they are believed to influence each other, see supplementary Fig. 2. As the HLI is a composite score of all behaviors measured, it could not be adjusted for individual behaviors and was only adjusted for sociodemographic and health factors. For both models, an interaction between participant’s sex and age group (above or below 75 years old at HUNT4) and their HLI was included.

The proportional odds assumption was assessed using the poTest function from the R library car v.3.1-1 [28], that is an implementation of the Brant test for assessing proportionality in the proportional odds model.

For individual factors of the HLI, being in the “high risk” behavior group was used as the reference category.

## Results

**Fig. 1:**
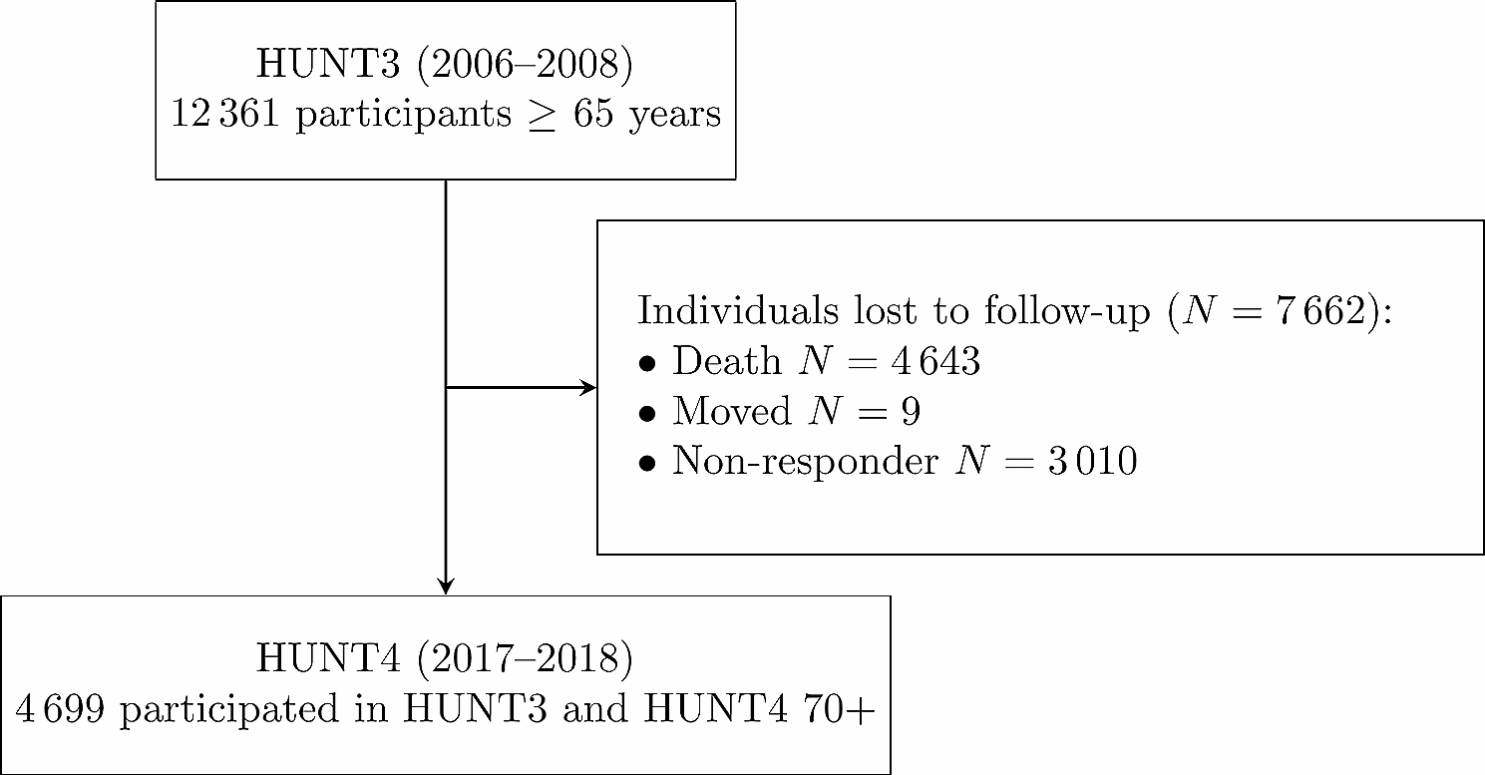
Flowchart over recruitment and loss to follow-up

There were 12,361 participants in HUNT 3 that were ≥ 65 years at time of participation. Of these, 4699 also participated in HUNT4 and HUNT4 70+. Of the 7662 that did not participate in HUNT4, 4652 had died or moved before HUNT4 was carried out. Details can be seen in Fig. 1.

### Descriptive statistics

In Tables 3 and 4 the proportion of participants in the individual risk and impairment categories for the HLI and ICI respectively are presented. Of the health-promoting behaviors measured in HUNT 3, the highest risk observed was from low fruit and vegetable intake (47.2%), low intake of milk (46.6%), and lack of physical activity (31.1%).

**Table 3:** Characteristics of individual health-promoting behaviors of participants in HUNT3

	**Risk from behavior**			**Percent imputed values**
	No/mild risk	Moderate risk	High risk	
**Physical activity**	2190 (46.6%)	1046 (22.3%)	1463 (31.1%)	2.3–19.6%
**Social interaction**	3742 (79.6%)	753 (16%)	204 (4.3%)	9.4%
**Smoking**	2259 (48.1%)	1860 (39.6%)	580 (12.3%)	4.1%
**Alcohol intake**	3348 (71.2%)	1079 (23%)	272 (5.8%)	10.9%
**Sleep quality**	3398 (72.3%)	1116 (23.7%)	185 (3.9%)	10.4–11.3%
**Fruit and vegetables intake**	231 (4.9%)	2248 (47.8%)	2220 (47.2%)	0.1%
**Fatty fish intake**	3932 (83.7%)	0 (0%)	767 (16.3%)	3.7%
**Choose low fat dairy**	2899 (61.7%)	1172 (24.9%)	628 (13.4%)	9.5–17.3%
**Milk low fat**	912 (19.4%)	1596 (34%)	2191 (46.6%)	9.5%
**Choose oils or soft margarine on bread or cooking**	4293 (91.4%)	0 (0%)	406 (8.6%)	9.4–12.3%

Note: The percent imputed values column show the percent of values that have been imputated. If multiple variables were used in the category, a range is shown

Of the individual domains of intrinsic capacity in HUNT4, most impairment was seen in the domains of locomotion (29.7%), hearing (11.0%) and vitality (12.0%).

**Table 4:** Characteristics of individual intrinsic capacity domains of participants in HUNT4

	Impairment category			Percent imputed values
	No/mild impairment	Moderate impairment	High impairment	
**Locomotion**	2252 (47.9%)	1053 (22.4%)	1394 (29.7%)	3.3%
**Cognition**	4033 (85.8%)	443 (9.4%)	223 (4.7%)	7%
**Vitality**	3363 (71.6%)	773 (16.5%)	563 (12%)	6.3%
**Mental health**	4140 (88.1%)	538 (11.4%)	21 (0.4%)	1.4%
**Hearing**	3544 (75.4%)	637 (13.6%)	518 (11%)	9.6%
**Vision**	4038 (85.9%)	462 (9.8%)	199 (4.2%)	9.6%

Note: The percent imputed values column show the percent of values that have been imputated

Characteristics of the participants based on their quartile score on the HLI and ICI are described in Table 5. Overall, the score on both the HLI and ICI was high. The median and interquartile range of the HLI was 6.5 (5.5–6.5), and the median and interquartile range of the ICI was 5.0 (4.5-6). Participants who scored above the median were more likely to have higher levels of education and be married. For intrinsic capacity lower capacity was more likely in the older age groups, while the HLI was similar across different ages. There were fewer individuals with three or more long-term conditions in the higher quartiles for both the HLI and the ICI, while the opposite trend was seen for individuals with no long-term conditions.

**Table 5:** Characteristics of the study sample according to healthy life index (HUNT3) and intrinsic capacity (HUNT4)

	Healthy life index HUNT3				Intrinsic capacity HUNT4			
	First quartile	Second quartile	Third quartile	Fourth quartile	First quartile	Second quartile	Third quartile	Fourth quartile
**Age, median (IQR)**								
Age	70.5 (67.1-74.8)	70.4 (67.4–75.2)	70.75 (67.3–75)	70.5 (67.4–74.8)	86.8 (82–90.7)	83.5 (79.7–88)	80.5 (77.8–84.1)	78.7 (76.8–81.3)
**Sex, counts (%)**								
Women	560 (57.9%)	730 (55.6%)	438 (55.9%)	927 (56.7%)	645 (60.7%)	314 (59.1%)	996 (56.3%)	700 (52.3%)
Men	408 (42.1%)	582 (44.4%)	346 (44.1%)	708 (43.3%)	417 (39.3%)	217 (40.9%)	772 (43.7%)	638 (47.7%)
**Marital status, counts (%)**								
Married	565 (58.4%)	881 (67.1%)	566 (72.2%)	1196 (73.1%)	584 (55%)	339 (63.8%)	1260 (71.3%)	1025 (76.6%)
Unmarried/Widow/Divorced/Separated	403 (41.6%)	431 (32.9%)	218 (27.8%)	439 (26.9%)	478 (45%)	192 (36.2%)	508 (28.7%)	313 (23.4%)
**Education level, counts (%)**								
Primary school	403 (41.6%)	461 (35.1%)	265 (33.8%)	525 (32.1%)	516 (48.6%)	221 (41.6%)	573 (32.4%)	344 (25.7%)
Vocational education	367 (37.9%)	506 (38.6%)	290 (37%)	590 (36.1%)	357 (33.6%)	211 (39.7%)	698 (39.5%)	487 (36.4%)
Upper secondary school	52 (5.4%)	98 (7.5%)	47 (6%)	105 (6.4%)	59 (5.6%)	27 (5.1%)	111 (6.3%)	105 (7.8%)
University/Community college less than four years	80 (8.3%)	142 (10.8%)	102 (13%)	222 (13.6%)	74 (7%)	51 (9.6%)	222 (12.6%)	199 (14.9%)
University/Community college four years or more	66 (6.8%)	105 (8%)	80 (10.2%)	193 (11.8%)	56 (5.3%)	21 (4%)	164 (9.3%)	203 (15.2%)
**Number of long term conditions**								
None	229 (23.7%)	340 (25.9%)	203 (25.9%)	527 (32.2%)	207 (19.5%)	108 (20.3%)	496 (28.1%)	488 (36.5%)
One condition	295 (30.5%)	409 (31.2%)	269 (34.3%)	492 (30.1%)	311 (29.3%)	164 (30.9%)	554 (31.3%)	436 (32.6%)
Two conditions	221 (22.8%)	303 (23.1%)	163 (20.8%)	355 (21.7%)	255 (24%)	119 (22.4%)	394 (22.3%)	274 (20.5%)
Three or more conditions	223 (23%)	260 (19.8%)	149 (19%)	261 (16%)	289 (27.2%)	140 (26.4%)	324 (18.3%)	140 (10.5%)

### The association of health promoting behaviors in older adulthood with intrinsic capacity 10 years later

Participants with a higher HLI also had a higher ICI score ten years later, and this held true after adjusting for age, sex, level of education, marital status and number of long-term conditions. A one-point increase in the HLI was associated with a 1.08 times increased chance of being in a higher ICI category. With the exception of alcohol and intake of dairy, being in the moderate and no/mild risk for the individual health-promoting behaviors were associated with improved ICI compared to being in the high-risk group. For the behaviors physical activity, social interaction and fruit and vegetable intake there is a potential dose-response as the benefit of being in the low-risk group is higher than being in the moderate risk group when compared with the high-risk group. The odds ratios and 95% confidence intervals for each model are presented in Table 6. The second model for physical activity did not fulfill the proportional odds assumption, and is not included in the table. No significant interaction was found between sex, age group and the HLI, so the interaction term is not included in the table.

**Table 6:** Ordinal logistic regression results. For all individual behaviors, high risk was used as the reference category

Model	Model 1^i^	Model 2^ii^
	Odds ratio (95% CI)	Odds ratio (95% CI)
**Healthy Life Index** ^**iii**^		
Healthy Life Index	1.08 (1.01;1.15)	
**Physical activity** ^**acde**^		
Moderate risk	1.24 (1.06;1.44)	1.24 (1.06;1.44)
No/mild risk	1.34 (1.17;1.53)	1.34 (1.17;1.53)
**Social Interaction**		
Moderate risk	1.4 (1.05;1.86)	1.4 (1.05;1.86)
No/mild risk	1.86 (1.42;2.43)	1.86 (1.42;2.43)
**Smoking** ^**b**^		
Moderate risk	1.19 (0.99;1.43)	1.19 (0.99;1.43)
No/mild risk	1.13 (0.94;1.36)	1.13 (0.94;1.36)
**Alcohol** ^**a**^		
Moderate risk	1.11 (0.85;1.45)	1.11 (0.85;1.45)
No/mild risk	1.02 (0.79;1.31)	1.02 (0.79;1.31)
**Sleep** ^**abc**^		
Moderate risk	1.07 (0.79;1.44)	1.07 (0.79;1.44)
No/mild risk	1.18 (0.88;1.56)	1.18 (0.88;1.56)
**Fruit and vegetables intake** ^**abcd**^		
Moderate risk	1.24 (1.1;1.39)	1.24 (1.1;1.39)
No/mild risk	1.4 (1.05;1.87)	1.4 (1.05;1.87)
**Fatty fish intake** ^**abcd**^		
No/mild risk	1.18 (1.01;1.37)	1.18 (1.01;1.37)
**Choose low fat dairy** ^**abcd**^		
Moderate risk	1.05 (0.86;1.26)	1.05 (0.86;1.26)
No/mild risk	1.07 (0.9;1.26)	1.07 (0.9;1.26)
**Milk low fat** ^**abcd**^		
Moderate risk	1 (0.88;1.14)	1 (0.88;1.14)
No/mild risk	0.88 (0.75;1.02)	0.88 (0.75;1.02)
**Choose oils or soft margarine on bread or cooking** ^**abcd**^		
No/mild risk	1.19 (0.98;1.45)	1.19 (0.98;1.45)

^i^Adjusted for: Age, sex, level of education, marital status and number of long-term conditions

^ii^Adjusted for: ^a^: Social interaction ^b^: Alcohol intake ^c^: Smoking status ^d^: Sleep quality ^e^: Diet quality

## Discussion

In this study we aimed to explore if adherence to health-promoting behaviors in older adulthood was associated with improved intrinsic capacity at ten year follow up. We found that even when controlling for known predictors of lower adherence to guidelines for healthy living and lower functional ability, closer adherence to guidelines for healthy living was associated with higher intrinsic capacity ten years later. These findings support the WHO’s ICOPE guidelines [8] that it is beneficial to take multiple health-promoting behaviors into account when working to improve an individual’s intrinsic capacity. Establishing these behaviors early may have additional benefits, as they are associated with reduced prevalence of long-term conditions that can lead to lower intrinsic capacity during ageing, shown in our data by the lower intrinsic capacity in the participants with long-term conditions. Reducing risk of non-communicable diseases and/or disease severity and improved metabolic health are likely causal mechanisms behind these findings.

Unsurprisingly, we found that adherence to fruit and vegetable intake and physical activity level guidelines was low. This is in line with dietary intake data from Norway which show that intake of fruit and vegetables have increased since the time of HUNT3 [29]. Also following trends seen in Norwegian diets, the intake of milk and fatty fish reported by our HUNT cohort is higher than more recent data in Norway showing a decreased intake of these foods in the years since HUNT3 [29]. Similarly, since HUNT3 an increase in physical activity levels has been reported in the Norwegian population, including in older adults [30].

Looking at the association between individual components of the HLI and intrinsic capacity, the strongest associations were found with social interaction, fruit and vegetable intake and physical activity levels. This is in line with a study by Jia et al. [11] that found that diet, physical activity and social contact were most strongly associated with memory function. For the other components of adherence to dietary guidelines, fatty fish intake and vegetable oils tends towards being a positive influence on intrinsic capacity, while dairy showed no such association. Both fatty fish and vegetable oils are strongly associated with improved health in multiple domains [31, 32], while the data on dairy is more mixed though in favor of consumption [33, 34]. The strongest association was seen for social interaction, which is likely due to people lacking social support networks having fewer resources to play on in the face of health problems, and that lonely people are more likely to demonstrate less health-promoting behaviors [35, 36]. Looking at multiple behaviors and multiple domains of intrinsic capacity together is important as individuals partake in multiple behaviors and can have multiple functional impairments. Seen together it is easier to observe benefits of health-promoting behavior for healthy ageing. Increasing knowledge of the benefits of health-promoting behaviors, while not minimising the consequences of non-adherence, has been suggested as a way to increase occurrence of health-promoting behaviors in the older population [37].

Unfortunately, we were not able to assess participants intake of other foods known to influence health, such as whole grains, red and processed meat, ultra-processed foods and other types of seafood than fatty fish as these food items were not assessed in HUNT3.

Tavassoli et al. [38] found highest levels of impairment in the domains of locomotion, cognition and vitality when assessing intrinsic capacity in primary care services in France. This is similar to our results that also found locomotion and vitality to be two of the three domains with most impairment. In our sample, hearing impairment was more prevalent than impairment in both vitality and cognition, which may be due to different methods of measuring these domains but also that individuals with cognitive impairments may be more prevalent in a primary care setting than in HUNT participants. However, when cognition was measured in HUNT4 70 + participants could have the assessment health care team come to their home, likely improving participation in the more impaired population. Still, our cohort that took part in both HUNT3 and HUNT4 70 + has a lower level of cognitive impairment than what was found for all participants in HUNT4 70+ [39], and this could be due to how repeat participants in cohort studies are known to have better health outcomes than non-responders [40]. In our study, the majority of non-responder were participants that died between the two HUNT surveys. These participants were generally older, and had more missing data on the variables included in the HLI.

The questions used to assess health-promoting behaviors were short and simple, indicating that even a short questionnaire on adherence to healthy behaviors may be adequate to assess an older individual’s health-promoting behaviors with the potential to improve their long-term intrinsic capacity. This could help design questionnaires to be used in primary care practice, with a low burden on both participants and clinicians. WHOs ICOPE screening regime for intrinsic capacity has already been shown to be feasible to use in clinical practice [38]. While the individual has a responsibility to manage their health, differences in living conditions and socioeconomic factors mean that not everyone has the same opportunities for good health. For best care, it is important for healthcare workers to be aware of the stigma that can arise if ill health is blamed on a lack of personal responsibility and an inability to live healthily [41].

### Strengths and limitations

Strengths of this study are the use of the HUNT cohort, with a large sample and long follow-up time; the multiple behaviors that compose our HLI; and that besides the sensory impairment and mental health domains of intrinsic capacity all domains were measured by validated tests which have been proposed to be used by clinicians to assess patients. There is a clear temporal association between the HLI and ICI variables as the HLI measurements were collected ten years prior to the ICI.

Limitations include the self-reported behavior data, especially as these have not been validated for our cohort’s age group, and that the HLI is dominated by dietary factors. Additionally, for the domain of sleep we were only able to look at participant’s symptoms of insomnia, which may be insufficient as a measure of sleep quality. Previous studies have found relationships between other markers of sleep quality, like self-reported sleep quality and hours spent in bed, and functional capacities in the elderly [42]. It is also possible that health-promoting behaviors have changed during the ten-year period between HUNT3 and HUNT4 70+. While we controlled our models for the presence of long-term conditions, the severity of the conditions was not assessed, and the participants may have suffered from additional health conditions not included in the questionnaires. Finally, at the time of HUNT3, some participants may already have lowered functional capacities due to ill health that make them less able to comply with health-promoting guidelines.

Our cohort is from a high-income country with a relatively good health status. However, when accounting for functional measures the burden of the ageing population may be similar to lower income countries, although occurring later in life [43]. The HLI encompasses public health challenges, such as low fruit and vegetable intake and low levels of physical activity, that are not isolated to high income countries [44, 45]. Therefore our findings may be applicable to different populations.

Still, it is likely that the participants in our cohort are healthier, more health aware and have a higher socioeconomic status than the general population [46]. This is indicated by the high HLI and ICI of our participants and may have affected our results. Health-promoting behaviors are associated with a longer lifespan, and older age is associated with more functional impairments which could lead to an underestimation of the effect of health-promoting behaviors on intrinsic capacity through reverse causality.

## Conclusion

Participating in multiple health-promoting behaviors was associated with intrinsic capacity ten years later. Behaviors with the strongest associations with their intrinsic capacity were consuming more plant-based foods and participating in physical activity. The measurements used for assessing the health-promoting behaviors were simple, showing that even short questionnaires may provide useful information to a clinician to assess their patients. This could be used to help design short questionnaires to be used in clinical practice for assessing health-promoting behaviors. Still, due to the design of the study, we cannot conclude that lower adherence to healthy guidelines is not due to already present functional impairments and this should be elucidated in further studies.

## Electronic supplementary material

Below is the link to the electronic supplementary material.

Supplementary Material 1

## Data Availability

HUNT data is stored in the HUNT databank, and to protect the privacy of participants the HUNT Research Centre aims to limit storage of the data outside this databank. The HUNT Research Centre can reproduce the data used in this project upon request, for more information see: http://www.ntnu.edu/hunt/data. All scripts to analyze the data and create Figures and Tables for this study are available from a Zenodo repository [47].
