# Correction: Health-promoting behaviors in older adulthood and intrinsic capacity 10 years later: the HUNT study

**DOI:** 10.1186/s12889-025-23672-6

**Published:** 2025-07-24

**Authors:** Aslaug Angelsen, Sigrid Nakrem, Ekaterina Zotcheva, Bjørn Heine Strand, Linn Beate Strand

**Affiliations:** 1 University of Science and Technology, Trondheim, Norway; 2 Norwegian National Centre for Ageing and Health, Tønsberg, Norway; 3 Norwegian Institute of Public Health, Oslo, Norway

**Correction: BMC Public Health 24**,** 284 (2024)**

**https://doi.org/10.1186/s12889-024-17840-3**

The data registry that supplied the data, HUNT databank, discovered an error in the grip strength variable used in our analysis to assess the “Vitality” domain of intrinsic capacity, that has affected about a third of the participants in our study. We have been provided with the corrected values and re-run all analyses accordingly. Tables 3, 4 and 5 have been updated in addition to a minor change in the regression model with the “healthy life index” as exposure where our “intrinsic capacity index” outcome was changed from a five-level factor to a four level factor to comply with the proportional odds assumption.

## Methods section

### Original 2nd paragraph of “Ordinal regression” subchapter

Intrinsic capacity was categorised into five groups based on the summary score; ≤ 2, 3, 4, 5 and 6, with the score being rounded upwards to the largest number before categorisation, i.e. 2.5 would be “3”, and 5.5 would be “6”. Scores 1–2 were combined as there were only 18 participants that scored between 0 and 1.5.

### Corrected 2nd paragraph of “Ordinal regression” subchapter

Intrinsic capacity was categorised into five groups based on the summary score; ≤ 2, 3, 4, 5 and 6, with the score being rounded upwards to the largest number before categorisation, i.e. 2.5 would be “3”, and 5.5 would be “6”. Scores 1–2 were combined as there were only 18 participants that scored between 0 and 1.5. For the model using the HLI as an exposure, intrinsic capacity was categorised into four groups; ≤ 2, 3, 4 and ≥ 5, to fulfill the proportional odds assumption. Scores 5–6 were combined as it was assumed that combining the least impaired participants would preserve the most information from the different levels in the scale.

## Results section

### Incorrect descriptive statistics

Of the health-promoting behaviors measured in HUNT 3, the highest risk observed was from low fruit and vegetable intake (47.2%), low intake of milk (46.7%), and lack of physical activity (31.1%).

Of the individual domains of intrinsic capacity in HUNT4, most impairment was seen in the domains of locomotion (29.7%), hearing (11.1%) and vitality (8.3%).

Overall, the score on both the HLI and ICI was high. The median and interquartile range of the HLI was 6.5 (5.5–6.5), and the median and interquartile range of the ICI was 5.5 (4.5-6).

### Correct descriptive statistics

Of the health-promoting behaviors measured in HUNT 3, the highest risk observed was from low fruit and vegetable intake (47.2%), low intake of milk **(46.6%)**, and lack of physical activity (31.1%).

Of the individual domains of intrinsic capacity in HUNT4, most impairment was seen in the domains of locomotion (29.7%), hearing **(11.0%)** and vitality **(12.0%)**.

Overall, the score on both the HLI and ICI was high. The median and interquartile range of the HLI was 6.5 (5.5–6.5), and the median and interquartile range of the ICI was 5.**0** (4.5-6).

### Incorrect association of health promoting behaviors in older adulthood with intrinsic capacity 10 years later

A one point increase in the HLI was associated with a 1.15 times increased chance of being in a higher ICI category.

### Correct association of health promoting behaviors in older adulthood with intrinsic capacity 10 years later

A one point increase in the HLI was associated with a 1.**08** times increased chance of being in a higher ICI category.

**Incorrect Table 3**

**Table:** 

	Risk from behavior			
	No/mild risk	Moderate risk	High risk	Percent imputed values
**Physical activity**	2195 (46.7%)	1043 (22.2%)	1461 (31.1%)	2.3–19.6%
**Social interaction**	3738 (79.5%)	757 (16.1%)	204 (4.3%)	9.4%
**Smoking**	2259 (48.1%)	1860 (39.6%)	580 (12.3%)	4.1%
**Alcohol intake**	3337 (71%)	1108 (23.6%)	254 (5.4%)	10.9%
**Sleep quality**	3398 (72.3%)	1114 (23.7%)	187 (4%)	10.4–11.3%
**Fruit and vegetables intake**	231 (4.9%)	2248 (47.8%)	2220 (47.2%)	0.1%
**Fatty fish intake**	3933 (83.7%)	0 (0%)	766 (16.3%)	3.7%
**Choose low fat dairy**	2892 (61.5%)	1177 (25%)	630 (13.4%)	9.5–17.3%
**Milk low fat**	917 (19.5%)	1588 (33.8%)	2194 (46.7%)	9.5%
**Choose oils or soft margarine on bread or cooking**	4292 (91.3%)	0 (0%)	407 (8.7%)	9.4–12.3%

Note: The percent imputed values column show the percent of values that have been imputated. If multiple variables were used in the category, a range is shown

**Correct Table 3**

**Table:** 

	Risk from behavior			
	No/mild risk	Moderate risk	High risk	Percent imputed values
**Physical activity**	2190 (46.6%)	1046 (22.3%)	1463 (31.1%)	2.3–19.6%
**Social interaction**	3742 (79.6%)	753 (16%)	204 (4.3%)	9.4%
**Smoking**	2259 (48.1%)	1860 (39.6%)	580 (12.3%)	4.1%
**Alcohol intake**	3348 (71.2%)	1079 (23%)	272 (5.8%)	10.9%
**Sleep quality**	3398 (72.3%)	1116 (23.7%)	185 (3.9%)	10.4–11.3%
**Fruit and vegetables intake**	231 (4.9%)	2248 (47.8%)	2220 (47.2%)	0.1%
**Fatty fish intake**	3932 (83.7%)	0 (0%)	767 (16.3%)	3.7%
**Choose low fat dairy**	2899 (61.7%)	1172 (24.9%)	628 (13.4%)	9.5–17.3%
**Milk low fat**	912 (19.4%)	1596 (34%)	2191 (46.6%)	9.5%
**Choose oils or soft margarine on bread or cooking**	4293 (91.4%)	0 (0%)	406 (8.6%)	9.4–12.3%

Note: The percent imputed values column show the percent of values that have been imputated. If multiple variables were used in the category, a range is shown

**Incorrect Table 4**

**Table:** 

	Impairment category			
	No/mild impairment	Moderate impairment	High impairment	Percent imputed values
**Locomotion**	2252 (47.9%)	1052 (22.4%)	1395 (29.7%)	3.3%
**Cognition**	4023 (85.6%)	473 (10.1%)	203 (4.3%)	7%
**Vitality**	3702 (78.8%)	606 (12.9%)	391 (8.3%)	6.3%
**Mental health**	4143 (88.2%)	535 (11.4%)	21 (0.4%)	1.4%
**Hearing**	3541 (75.4%)	638 (13.6%)	520 (11.1%)	9.6%
**Vision**	4040 (86%)	468 (10%)	191 (4.1%)	9.6%

Note: The percent imputed values column show the percent of values that have been imputated

**Correct Table 4**

**Table:** 

	Impairment category			
	No/mild impairment	Moderate impairment	High impairment	Percent imputed values
**Locomotion**	2252 (47.9%)	1053 (22.4%)	1394 (29.7%)	3.3%
**Cognition**	4033 (85.8%)	443 (9.4%)	223 (4.7%)	7%
**Vitality**	3363 (71.6%)	773 (16.5%)	563 (12%)	6.3%
**Mental health**	4140 (88.1%)	538 (11.4%)	21 (0.4%)	1.4%
**Hearing**	3544 (75.4%)	637 (13.6%)	518 (11%)	9.6%
**Vision**	4038 (85.9%)	462 (9.8%)	199 (4.2%)	9.6%

Note: The percent imputed values column show the percent of values that have been imputated.

**Incorrect Table 5**

**Table:** 

	Healthy life index HUNT3				Intrinsic capacity HUNT4			
	First quartile	Second quartile	Third quartile	Fourth quartile	First quartile	Second quartile	Third quartile	Fourth quartile
**Age, median (IQR)**								
Age	70.6 (67.1–74.9)	70.4 (67.4–75.2)	70.5 (67.3–74.7)	70.5 (67.4–74.9)	86.6 (82-90.6)	82.3 (78.8–86.9)	80.1 (77.5–83.6)	78.7 (76.8–81.3)
**Sex**,** counts (%)**								
Women	568 (58.1%)	723 (55.9%)	433 (55.1%)	931 (56.7%)	581 (60.1%)	814 (59.2%)	534 (55.2%)	726 (52.3%)
Men	410 (41.9%)	570 (44.1%)	353 (44.9%)	711 (43.3%)	386 (39.9%)	562 (40.8%)	434 (44.8%)	662 (47.7%)
**Marital status**,** counts (%)**								
Married	572 (58.5%)	865 (66.9%)	573 (72.9%)	1197 (72.9%)	536 (55.4%)	899 (65.3%)	704 (72.7%)	1068 (76.9%)
Unmarried/Widow/Divorced/Separated	406 (41.5%)	428 (33.1%)	213 (27.1%)	445 (27.1%)	431 (44.6%)	477 (34.7%)	264 (27.3%)	320 (23.1%)
**Education level**,** counts (%)**								
Primary school	407 (41.6%)	460 (35.6%)	262 (33.3%)	523 (31.9%)	455 (47.1%)	541 (39.3%)	296 (30.6%)	360 (25.9%)
Vocational education	368 (37.6%)	496 (38.4%)	293 (37.3%)	598 (36.4%)	335 (34.6%)	526 (38.2%)	388 (40.1%)	506 (36.5%)
Upper secondary school	54 (5.5%)	95 (7.3%)	48 (6.1%)	105 (6.4%)	56 (5.8%)	70 (5.1%)	67 (6.9%)	109 (7.9%)
University/Community college less than four years	83 (8.5%)	137 (10.6%)	102 (13%)	224 (13.6%)	68 (7%)	136 (9.9%)	136 (14%)	206 (14.8%)
University/Community college four years or more	66 (6.7%)	105 (8.1%)	81 (10.3%)	192 (11.7%)	53 (5.5%)	103 (7.5%)	81 (8.4%)	207 (14.9%)
**Number of long term conditions**								
None	232 (23.7%)	333 (25.8%)	204 (26%)	528 (32.2%)	189 (19.5%)	326 (23.7%)	288 (29.8%)	494 (35.6%)
One condition	298 (30.5%)	403 (31.2%)	268 (34.1%)	501 (30.5%)	290 (30%)	404 (29.4%)	320 (33.1%)	456 (32.9%)
Two conditions	220 (22.5%)	301 (23.3%)	165 (21%)	350 (21.3%)	225 (23.3%)	326 (23.7%)	203 (21%)	282 (20.3%)
Three or more conditions	228 (23.3%)	256 (19.8%)	149 (19%)	263 (16%)	263 (27.2%)	320 (23.3%)	157 (16.2%)	156 (11.2%)

**Correct Table 5**

**Table:** 

	Healthy life index HUNT3				Intrinsic capacity HUNT4			
	First quartile	Second quartile	Third quartile	Fourth quartile	First quartile	Second quartile	Third quartile	Fourth quartile
**Age, median (IQR)**								
Age	70.5 (67.1–74.8)	70.4 (67.4–75.2)	70.75 (67.3–75)	70.5 (67.4–74.8)	86.8 (82–90.7)	83.5 (79.7–88)	80.5 (77.8–84.1)	78.7 (76.8–81.3)
**Sex**,** counts (%)**								
Women	560 (57.9%)	730 (55.6%)	438 (55.9%)	927 (56.7%)	645 (60.7%)	314 (59.1%)	996 (56.3%)	700 (52.3%)
Men	408 (42.1%)	582 (44.4%)	346 (44.1%)	708 (43.3%)	417 (39.3%)	217 (40.9%)	772 (43.7%)	638 (47.7%)
**Marital status**,** counts (%)**								
Married	565 (58.4%)	881 (67.1%)	566 (72.2%)	1196 (73.1%)	584 (55%)	339 (63.8%)	1260 (71.3%)	1025 (76.6%)
Unmarried/Widow/Divorced/Separated	403 (41.6%)	431 (32.9%)	218 (27.8%)	439 (26.9%)	478 (45%)	192 (36.2%)	508 (28.7%)	313 (23.4%)
**Education level**,** counts (%)**								
Primary school	403 (41.6%)	461 (35.1%)	265 (33.8%)	525 (32.1%)	516 (48.6%)	221 (41.6%)	573 (32.4%)	344 (25.7%)
Vocational education	367 (37.9%)	506 (38.6%)	290 (37%)	590 (36.1%)	357 (33.6%)	211 (39.7%)	698 (39.5%)	487 (36.4%)
Upper secondary school	52 (5.4%)	98 (7.5%)	47 (6%)	105 (6.4%)	59 (5.6%)	27 (5.1%)	111 (6.3%)	105 (7.8%)
University/Community college less than four years	80 (8.3%)	142 (10.8%)	102 (13%)	222 (13.6%)	74 (7%)	51 (9.6%)	222 (12.6%)	199 (14.9%)
University/Community college four years or more	66 (6.8%)	105 (8%)	80 (10.2%)	193 (11.8%)	56 (5.3%)	21 (4%)	164 (9.3%)	203 (15.2%)
**Number of long term conditions**								
None	229 (23.7%)	340 (25.9%)	203 (25.9%)	527 (32.2%)	207 (19.5%)	108 (20.3%)	496 (28.1%)	488 (36.5%)
One condition	295 (30.5%)	409 (31.2%)	269 (34.3%)	492 (30.1%)	311 (29.3%)	164 (30.9%)	554 (31.3%)	436 (32.6%)
Two conditions	221 (22.8%)	303 (23.1%)	163 (20.8%)	355 (21.7%)	255 (24%)	119 (22.4%)	394 (22.3%)	274 (20.5%)
Three or more conditions	223 (23%)	260 (19.8%)	149 (19%)	261 (16%)	289 (27.2%)	140 (26.4%)	324 (18.3%)	140 (10.5%)

**Incorrect Table 6**

**Table:** 

	Model 1	Model 2
Model	Odds ratio (95% CI)	Odds ratio (95% CI)
**Healthy Life Index**		
Healthy Life Index	1.15 (1.1;1.21)	
**Physical activity**		
Moderate risk	1.26 (1.08;1.47)	
No/mild risk	1.33 (1.16;1.51)	
**Social Interaction**		
Moderate risk	1.39 (1.04;1.85)	1.39 (1.04;1.85)
No/mild risk	1.94 (1.48;2.53)	1.94 (1.48;2.53)
**Smoking**		
Moderate risk	1.19 (0.99;1.43)	1.2 (1;1.44)
No/mild risk	1.21 (1.01;1.44)	1.22 (1.01;1.46)
**Alcohol**		
Moderate risk	1.11 (0.84;1.47)	1.11 (0.84;1.47)
No/mild risk	1.04 (0.8;1.35)	1.05 (0.8;1.36)
**Sleep**		
Moderate risk	1.11 (0.82;1.5)	1.08 (0.8;1.45)
No/mild risk	1.24 (0.93;1.65)	1.18 (0.89;1.57)
**Fruit and vegetables intake**		
Moderate risk	1.26 (1.12;1.42)	1.23 (1.09;1.39)
No/mild risk	1.45 (1.09;1.94)	1.43 (1.08;1.92)
**Fatty fish intake**		
No/mild risk	1.17 (1;1.36)	1.14 (0.98;1.33)
**Choose low fat dairy**		
Moderate risk	0.94 (0.78;1.14)	0.92 (0.76;1.12)
No/mild risk	1.02 (0.86;1.21)	1 (0.84;1.18)
**Milk low fat**		
Moderate risk	1.02 (0.89;1.16)	1 (0.88;1.14)
No/mild risk	0.91 (0.78;1.06)	0.9 (0.77;1.05)
**Choose oils or soft margarine on bread or cooking**		
No/mild risk	1.15 (0.94;1.4)	1.16 (0.95;1.41)

^i^Adjusted for: Age, sex, level of education, marital status and number of long-term conditions.

^ii^Adjusted for: ^a^: Social interaction^b^: Alcohol intake^c^: Smoking status^d^: Sleep quality^e^: Diet quality

**Correct Table 6**

**Table:** 

	Model 1^i^	Model 2^ii^
Model	Odds ratio (95% CI)	Odds ratio (95% CI)
**Healthy Life Index** ^iii^		
Healthy Life Index	1.08 (1.01;1.15)	
**Physical activity** ^**acde**^		
Moderate risk	1.24 (1.06;1.44)	1.24 (1.06;1.44)
No/mild risk	1.34 (1.17;1.53)	1.34 (1.17;1.53)
**Social Interaction**		
Moderate risk	1.4 (1.05;1.86)	1.4 (1.05;1.86)
No/mild risk	1.86 (1.42;2.43)	1.86 (1.42;2.43)
**Smoking** ^**b**^		
Moderate risk	1.19 (0.99;1.43)	1.19 (0.99;1.43)
No/mild risk	1.13 (0.94;1.36)	1.13 (0.94;1.36)
**Alcohol** ^**a**^		
Moderate risk	1.11 (0.85;1.45)	1.11 (0.85;1.45)
No/mild risk	1.02 (0.79;1.31)	1.02 (0.79;1.31)
**Sleep** ^**abc**^		
Moderate risk	1.07 (0.79;1.44)	1.07 (0.79;1.44)
No/mild risk	1.18 (0.88;1.56)	1.18 (0.88;1.56)
**Fruit and vegetables intake** ^**abcd**^		
Moderate risk	1.24 (1.1;1.39)	1.24 (1.1;1.39)
No/mild risk	1.4 (1.05;1.87)	1.4 (1.05;1.87)
**Fatty fish intake** ^**abcd**^		
No/mild risk	1.18 (1.01;1.37)	1.18 (1.01;1.37)
**Choose low fat dairy** ^**abcd**^		
Moderate risk	1.05 (0.86;1.26)	1.05 (0.86;1.26)
No/mild risk	1.07 (0.9;1.26)	1.07 (0.9;1.26)
**Milk low fat** ^**abcd**^		
Moderate risk	1 (0.88;1.14)	1 (0.88;1.14)
No/mild risk	0.88 (0.75;1.02)	0.88 (0.75;1.02)
**Choose oils or soft margarine on bread or cooking** ^**abcd**^		
No/mild risk	1.19 (0.98;1.45)	1.19 (0.98;1.45)

^i^Adjusted for: Age, sex, level of education, marital status and number of long-term conditions.

^ii^Adjusted for: ^a^: Social interaction^b^: Alcohol intake^c^: Smoking status^d^: Sleep quality^e^: Diet quality

^iii^To fulfill the proportional odds assumption, the intrinsic capacity outcome is coded as a four level variable in this model, compared to a five level model in the others.

The original article [1] has been updated.
