# Supplementary material for: Health-promoting behaviors in older adulthood and intrinsic capacity 10 years later: the HUNT study
Source: BMC Public Health. 2024 Jan 24;24:284. doi: 10.1186/s12889-024-17840-3 (PMC10809656; doi:10.1186/s12889-024-17840-3)
Supplement: Supplementary file 1 — Supplementary Material 1 [file 12889_2024_17840_MOESM1_ESM.docx]

# Health-promoting behaviors in older adulthood and intrinsic capacity 10 years later: The HUNT study - Supplementary files

## Healthy life index

### Physical activity

Participants were asked about the mean frequency, intensity and duration of their weekly leisure physical activity. Response options for frequency were “never”, “less than once a week”, “once a week”, “2-3 times a week” and “about every day”. These responses were recoded as 0, 1, 2.5 and 5 days respectively. Response options for intensity were “I take it slow without becoming breathless or sweaty”, “I become breathless and sweaty” and “I exhaust myself”. The first response was recoded into “moderate” intensity, and the two others to “vigorous” activity. Response options for duration were “less than 15 minutes”, “15 - 29 minutes”, “30 minutes - 1 hour” and “more than one hour”, recoded to “7.5”, “22.5”, “45” and “60” minutes of activity. A similar recoding scheme has been used previously [[1](#ref-kieffer2021)]. The recommendation in Norway is to achieve ≥ 150 minutes of moderate or ≥ 75 minutes of vigorous activity a week, or a combination of the two [[2](#ref-NordicCouncilofMinisters2014)]. To calculate the minutes a week of moderate activity, number of days were multiplied with duration in minutes and level of intensity. One minute of vigorous activity was considered equal to two minutes of moderate activity.

A categorical variable with three levels of “low risk”, “moderate risk” and “high risk” was made to show the risk of adverse health associated with participants adherence to physical activity guidelines. “Low risk” was considered ≥ 150 minutes moderate activity/week, “moderate risk” as ≥ 75 to < 150 minutes moderate activity/week, and “high risk” as <75 minutes moderate activity/week.

### Social interaction

Participants were asked “have you felt lonely in the last two weeks?” With response options being “no”, “a little”, “a good amount” and “often”. Answering “no” was considered “low risk”, “a little” as “moderate risk” and answering “a good amount” or “often” as “high risk”.

### Smoking

For smoking participants answered if they were “never smokers”, “former smokers” or “current smokers”. A three level variable was made with never smokers considered “low risk”, former smokers at “moderate risk” and current smokers at “high risk”.

### Alcohol intake

For alcohol intake (not included in the summary score), participants recorded the number of units of alcohol consumed in the last week. An intake of ≤ 2 two units of alcohol/week was defined as low-risk, an intake of 3-6 defined as “moderate risk” and an intake of ≥ 7 was defined as “high risk” [[4](#ref-anderson2023)].

### Sleep quality

For sleep quality, participants were asked about the presence of insomnia at morning, evening or night. Response categories were “never/almost never”, “sometimes” and “often”. A categorical variable was created, where participants that answered “never/almost never” or “sometimes” to all time-points were considered “low risk”, experiencing insomnia “often” at one or two timepoints were considered “moderate risk” and experiencing insomnia morning, evening and night to be “high risk”.

### Dietary factors

Norwegian food based dietary guidelines contain recommendations for the frequency of intake of milk, fatty fish, and fruit and vegetables, that vegetable sources of fat should be preferred over animal sources and that low fat animal products should be preferred [[2](#ref-NordicCouncilofMinisters2014)].

For intake of milk, participants were asked the following question “How many glasses do you usually drink?” with the response options “1-6 glasses a week”, “1 glass a day”, “2-3 glasses a day” and “4 glasses or more a day”. Individual questions were asked for “whole milk” and “other milk”. For intake of fatty fish, fruit and vegetables participants were asked “how often do you normally eat these foods?” with response options “0-3 times a month”, “1-3 times a week”, “4-6 times a week”, “once a day” and “twice or more a day”. For source of fat, participants were asked “what type of fat do you use on bread/when cooking?” with response options “butter”, “hard margarine”, “soft margarine”, “oils” and “do not use”. For frequency of intake responses were recoded into daily (milk, fruit and vegetables) or weekly (fatty fish) frequencies as done previously by Mostad et al. [[5](#ref-mostad2014)], to be in line with Norwegian food based dietary guidelines for these foods [[2](#ref-NordicCouncilofMinisters2014)]. A “low risk” intake was defined as choosing low fat milks over whole fat milk; oils and soft margarine over butter and hard margarine for both bread and cooking; and a frequency of intake of ≥ two glasses of milk/day, ≥ two servings/week of fatty fish and ≥ four portions/day for fruit and vegetables, as to confirm as closely as possible with adherence to the Norwegian food based dietary guidelines [[2](#ref-NordicCouncilofMinisters2014)]. “Moderate risk” was defined as having an equal same intake of low and whole fat milk; choosing butter or margarine for either bread or cooking; and a frequency of intake of ≥ one to < two glasses of milk/day, ≥ one to < two servings/week of fatty fish and ≥ two to < four portions/day for fruit and vegetables. “High risk” was defined as drinking more whole fat than low milk; choosing butter or margarine for both bread or cooking; and a frequency of intake of < one glass of milk/day, < one serving/week of fatty fish and < two portions/day for fruit and vegetables.

## Intrinsic capacity index

### Locomotion

Locomotion was assessed by the participants’ scores on the Short Physical Performance Battery (SPPB). The SPPB was conducted either at a testing station, or by visits from ambulatory teams. The SPPB is comprised of three subtests that evaluates balance, gait, strength and endurance: a timed hierarchic balance test, a 4-m gait speed test and a repeated sit-to-stand test. Performance on each subtest is scored from 0-4, with 0 being unable to perform the test and 4 showing no impairment, giving a maximum score of 12 for all subtests [[6](#ref-bergland2019)].

Thresholds for level of functioning in the Norwegian translation of the SPPB were used to categorize the physical capacities of the participants [[7](#ref-bergh2013)]: 0-6 = High impairment; 7-9 = Moderate impairment; 10-12 = No/mild impairment.

### Cognition

Cognition was assessed by using the participants’ scores on the Montreal Cognitive Assessment (MoCA). MoCA is a global cognitive function test that consists of 10 subtests, testing visuospatial abilities, executive functioning, phonemic fluency, verbal abstraction, attention, concentration, working memory, language and orientation. The maximum score for MoCA is 30 while the lowest is 0. A higher score indicates a higher level of functioning.

Cut-offs for “high” and “moderate” impairment was <-2 and <-1 standard deviation respectively, based on participants’ age group, sex and level of education [[8](#ref-borland2017)]. Participants that scored above the cut-off were defined as having no or mild impairment.

### Vitality

To assess vitality, the mean grip strength of three tests of the dominant hand was used. The following thresholds were used to assess impairment was used: < 16,0 kg (female) / < 26,0 kg (male) = High impairment; 16,0–19,9 kg (female) / 26,0–31,9 kg (male) = Moderate impairment; ≥ 20 kg (female) / ≥ 32 kg (male) = No impairment. These threshold values have been used in the Norwegian population previously [[9](#ref-krogseth2021)].

### Psychology

Participants psychological well-being was assessed by the question “think about how your life at the moment, would you say that you by and large are satisfied with life, or are you mostly dissatisfied?” with response options “very dissatisfied”, “dissatisfied”, “somewhat dissatisfied”, “neither satisfied or dissatisfied”, “somewhat satisfied”, “satisfied” and “very satisfied”.

The answer categories were turned into a numeric variable from 1-7 with a higher score meaning a higher satisfaction with life. The following thresholds were used to categorize psychological functioning: ≤ 2 = High impairment; ≤ 4 = Moderate impairment; ≥ 5 = No/mild impairment.

### Sensory impairment

For sensory capacity, participants were first asked “do you suffer from long-term (at least 1 year) illness or injury of a physical or psychological nature that impairs your functioning in your daily life?” with response options “yes” or “no”. Participants that answered “yes” were further asked to rank both their visual and hearing function as being either “no impairment”, “mild impairment”, “moderate impairment” or “high impairment”.

Participants self-reported impairment was used to define level of impairment.

# Directed Acyclic Graph

A Directed Acyclic Graph (DAG) was constructed to visualise the assumed associations between the HLI, intrinsic capacity and important potential confounders, see S Figure 1.


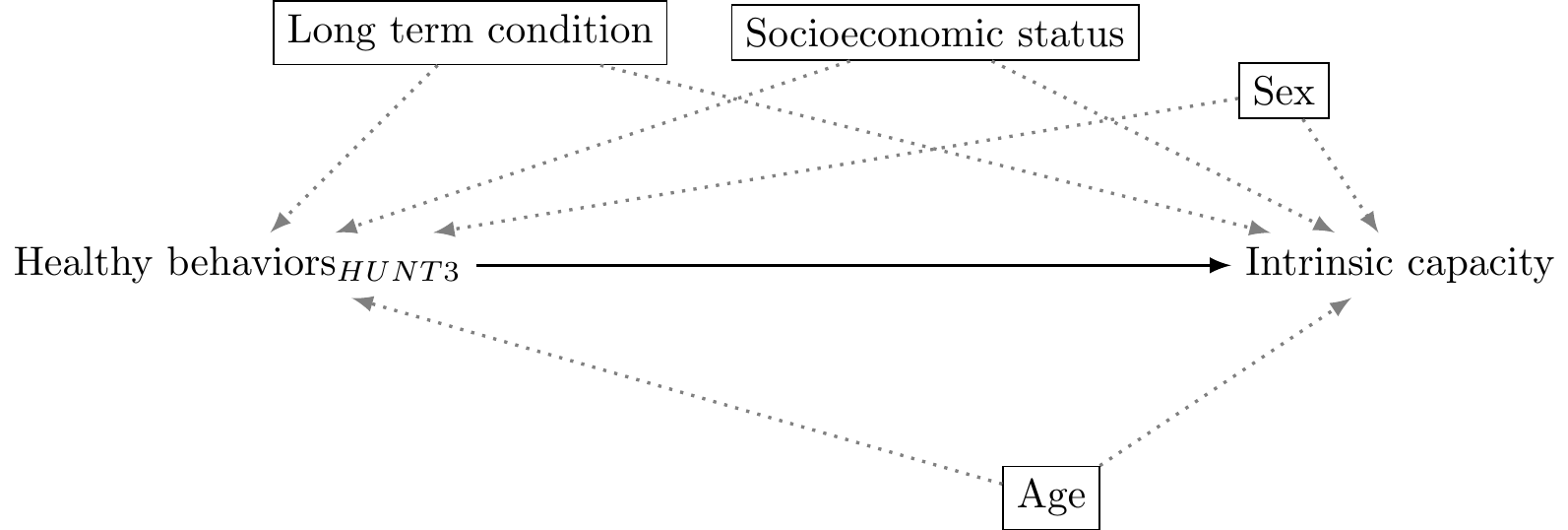


S Figure 1: Directed acyclic graph of the assumed associations between variables. Dashed lines indicate relationships that could go in either direction, the arrow points towards the direction we think is most important in this study. Boxed rectangles indicate potential confounders.

A more complex DAG was built for the associations of individual health-promoting behaviors, shown in S Figure 2. While intrinsic capacity was measured at the time of HUNT4, it is possible participant’s intrinsic capacity at the time of HUNT3 influenced behaviors at that timepoint, thus intrinsic capacity is indicated with a potential bidirectional relationship to the behaviors measured in HUNT3.


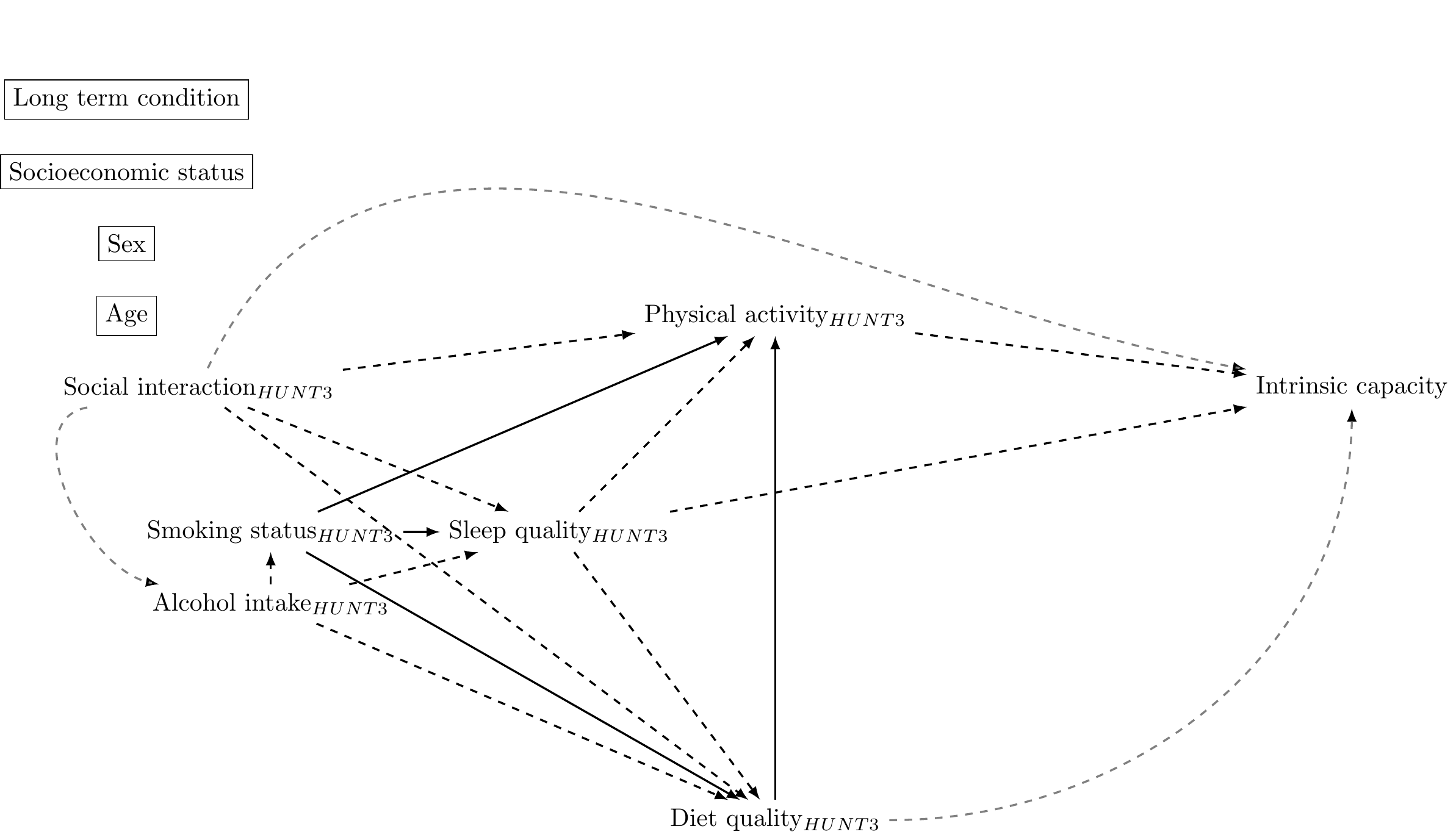


S Figure 2: Directed acyclic graph of the assumed associations between individual health-promoting behaviors. Dashed lines indicate relationships that could go in either direction, the arrow points towards the direction we think is most important in this study. Boxed rectangles indicate potential confounders.

S Table 1: References for the arrows made in the directed acyclic graphs.

| Arrow | Reference | Notes |
| --- | --- | --- |
| Loneliness Diet quality | [[10](#ref-besora-moreno2020)]; [[11](#ref-poggiogalle2021)]; [[12](#ref-abel2020)] | Potential overlap between loneliness/lack of social contact and depression in [[10](#ref-besora-moreno2020)]. Abel et al. [[12](#ref-abel2020)] is a report with Norwegian data. |
| Loneliness Alcohol intake | [[13](#ref-kelly2018)] | Loneliness brought up as a reason for drinking, but also that alcohol intake happens with social interactions. U relationship. Reference also briefly mentions differences between men and women and how having a long term condition can influence alcohol intake. |
| Loneliness Physical activity | [[14](#ref-hawkley2009)]; [[15](#ref-schrempft2019)] | [[15](#ref-schrempft2019)] found association between social isolation and physical activity, but not with loneliness. |
| Loneliness Sleep quality | [[16](#ref-griffin2020)] | Stronger association for social isolation and sleep than loneliness and sleep. The assoication with loneliness attenuates when depression state is taken into account. No association with gender or age. |
| Smoking Diet quality | [[17](#ref-alkerwi2017)] |  |
| Smoking Physical activity | [[18](#ref-kaczynski2008)] | NB, differences seen between age groups and sexes. |
| Smoking Sleep | [[19](#ref-hu2021)] |  |
| Sleep Physical activity | [[20](#ref-lambiase2013)]; [[21](#ref-berninger2020)]; [[22](#ref-baron2013)] |  |
| Sleep Diet quality | [[23](#ref-alkhatib2017)]; [[24](#ref-greer2013)] |  |
| Sleep Waist/Hip ratio | [[25](#ref-peltzer2017)] | [[25](#ref-peltzer2017)] looked at adults, not elderly. [[26](#ref-kakazu2022)] found no association between sleep quality and waist/hip ratio for eldery. |
| Physical activity Diet quality |  | Higher levels of physical activity, higher food intake and more likely to meet dietary needs. Appetite regulating effects of physical activity. |
| Behavior risk factors Disability old age | [[27](#ref-chakravarty2012)] |  |
| Socioeconomic status Diet quality | [[10](#ref-besora-moreno2020)] |  |

# References

1. Kieffer SK, Nauman J, Syverud K, Selboskar H, Lydersen S, Ekelund U, et al. [Association between Personal Activity Intelligence (PAI) and body weight in a population free from cardiovascular disease – The HUNT study](https://doi.org/10.1016/j.lanepe.2021.100091). The Lancet Regional Health – Europe. 2021;5.

2. Nordic Council of Ministers NC of M. [Nordic nutrition recommendations 2012](https://doi.org/10.6027/Nord2014-002). In: Nordic nutrition recommendations 2012. 5th edition. Copenhagen: Nordisk Ministerråd; 2014. p. 1–627.

3. Street A. Canada’s Guidance on Alcohol and Health: Final Report. Canada Centre of Substance use and Addiction; 2023.

4. Anderson BO, Berdzuli N, Ilbawi A, Kestel D, Kluge HP, Krech R, et al. [Health and cancer risks associated with low levels of alcohol consumption](https://doi.org/10.1016/S2468-2667(22)00317-6). The Lancet Public Health. 2023;8:e6–7.

5. Mostad IL, Langaas M, Grill V. [Central obesity is associated with lower intake of whole-grain bread and less frequent breakfast and lunch: Results from the HUNT study, an adult all-population survey](https://doi.org/10.1139/apnm-2013-0356). Appl Physiol Nutr Metab. 2014;39:819–28.

6. Bergland A, Strand BH. [Norwegian reference values for the Short Physical Performance Battery (SPPB): The Tromsø Study](https://doi.org/10.1186/s12877-019-1234-8). BMC Geriatrics. 2019;19:216.

7. Bergh S, Lyshol H, Selbæk G, Strand BH, Taraldsen K, Thingstad P. [Short Physical Performance Battery](https://www.legeforeningen.no/contentassets/870420284b7d4cb98100191ff93e7983/sppb.pdf). 2013.

8. Borland E, Nägga K, Nilsson PM, Minthon L, Nilsson ED, Palmqvist S. [The Montreal Cognitive Assessment: Normative Data from a Large Swedish Population-Based Cohort](https://doi.org/10.3233/JAD-170203). J Alzheimers Dis. 2017;59:893–901.

9. Krogseth M, Rostoft S, Benth JŠ, Selbæk G, Wyller TB. Skrøpelighet blant eldre pasienter med hjemmesykepleie. Tidsskrift for Den norske legeforening. 2021. <https://doi.org/10.4045/tidsskr.20.0688>.

10. Besora-Moreno M, Llauradó E, Tarro L, Solà R. [Social and Economic Factors and Malnutrition or the Risk of Malnutrition in the Elderly: A Systematic Review and Meta-Analysis of Observational Studies](https://doi.org/10.3390/nu12030737). Nutrients. 2020;12:737.

11. Poggiogalle E, Kiesswetter E, Romano M, Saba A, Sinesio F, Polito A, et al. [Psychosocial and cultural determinants of dietary intake in community-dwelling older adults: A Determinants of Diet and Physical Activity systematic literature review](https://doi.org/10.1016/j.nut.2020.111131). Nutrition. 2021;85:111131.

12. Abel MH. Resultater fra Den nasjonale folkehelseundersøkelsen 2020. 2020.

13. Kelly S, Olanrewaju O, Cowan A, Brayne C, Lafortune L. [Alcohol and older people: A systematic review of barriers, facilitators and context of drinking in older people and implications for intervention design](https://doi.org/10.1371/journal.pone.0191189). PLoS One. 2018;13:e0191189.

14. Hawkley LC, Thisted RA, Cacioppo JT. [Loneliness Predicts Reduced Physical Activity: Cross-Sectional & Longitudinal Analyses](https://doi.org/10.1037/a0014400). Health Psychol. 2009;28:354–63.

15. Schrempft S, Jackowska M, Hamer M, Steptoe A. [Associations between social isolation, loneliness, and objective physical activity in older men and women](https://doi.org/10.1186/s12889-019-6424-y). BMC Public Health. 2019;19:74.

16. Griffin SC, Williams AB, Ravyts SG, Mladen SN, Rybarczyk BD. [Loneliness and sleep: A systematic review and meta-analysis](https://doi.org/10.1177/2055102920913235). Health Psychol Open. 2020;7:2055102920913235.

17. Alkerwi A, Baydarlioglu B, Sauvageot N, Stranges S, Lemmens P, Shivappa N, et al. [Smoking status is inversely associated with overall diet quality: Findings from the ORISCAV-LUX study](https://doi.org/10.1016/j.clnu.2016.08.013). Clinical Nutrition. 2017;36:1275–82.

18. Kaczynski AT, Manske SR, Mannell RC, Grewal K. [Smoking and physical activity: A systematic review](https://doi.org/10.5555/ajhb.2008.32.1.93). Am J Health Behav. 2008;32:93–110.

19. Hu N, Wang C, Liao Y, Dai Q, Cao S. [Smoking and incidence of insomnia: A systematic review and meta-analysis of cohort studies](https://doi.org/10.1016/j.puhe.2021.07.012). Public Health. 2021;198:324–31.

20. Lambiase MJ, Gabriel KP, Kuller LH, Matthews KA. [Temporal relationships between physical activity and sleep in older women](https://doi.org/10.1249/MSS.0b013e31829e4cea). Med Sci Sports Exerc. 2013;45:2362–8.

21. Berninger N, Knell G, Gabriel KP, Plasqui G, Crutzen R, Hoor GT. [Bidirectional Day-to-Day Associations of Reported Sleep Duration With Accelerometer Measured Physical Activity and Sedentary Time Among Dutch Adolescents: An Observational Study](https://doi.org/10.1123/jmpb.2020-0010). J Meas Phys Behav. 2020;3:304–14.

22. Baron KG, Reid KJ, Zee PC. [Exercise to improve sleep in insomnia: Exploration of the bidirectional effects](https://doi.org/10.5664/jcsm.2930). J Clin Sleep Med. 2013;9:819–24.

23. Al Khatib HK, Harding SV, Darzi J, Pot GK. [The effects of partial sleep deprivation on energy balance: A systematic review and meta-analysis](https://doi.org/10.1038/ejcn.2016.201). Eur J Clin Nutr. 2017;71:614–24.

24. Greer SM, Goldstein AN, Walker MP. [The impact of sleep deprivation on food desire in the human brain](https://doi.org/10.1038/ncomms3259). Nat Commun. 2013;4:2259.

25. Peltzer K, Pengpid S. [Sleep Duration, Sleep Quality, Body Mass Index, and Waist Circumference among Young Adults from 24 Low- and Middle-Income and Two High-Income Countries](https://doi.org/10.3390/ijerph14060566). Int J Environ Res Public Health. 2017;14:566.

26. Kakazu VA, Pinto RZ, Dokkedal-Silva V, Fernandes GL, Gobbi C, Andersen ML, et al. [Sleep Quality, Body Mass Index and Waist-to-Hip Ratio in Older Adults](https://doi.org/10.1080/02703181.2021.1994098). Physical & Occupational Therapy In Geriatrics. 2022;40:150–60.

27. Chakravarty EF, Hubert HB, Krishnan E, Bruce BB, Lingala VB, Fries JF. [Lifestyle risk factors predict disability and death in healthy aging adults](https://doi.org/10.1016/j.amjmed.2011.08.006). The American Journal of Medicine. 2012;125:190–7.
